# Distribution and associated factors of choroidal thickness in highly myopic eyes—a real-world study based on a Chinese population

**DOI:** 10.1038/s41433-024-03383-9

**Published:** 2024-10-24

**Authors:** Lei Shao, HanQing Zhao, RuiHeng Zhang, WenDa Zhou, Wen Bin Wei

**Affiliations:** Beijing Tongren Eye Center, Beijing Tongren Hospital, Capital Medical University, Beijing, China

**Keywords:** Retinal diseases, Refractive errors

## Abstract

**Purpose:**

To measure the subfoveal choroidal thickness (SFCT) in highly myopic eyes at different locations using enhanced depth imaging spectral-domain optical coherence tomography (EDI SD-OCT). To identify the ocular and systemic risk factors associated with choroidal thinning in high myopia.

**Methods:**

Based on the Beijing Eye Study, a detailed ophthalmic examination was performed including EDI SD-OCT for the measurement of SFCT. OCT images were obtained from 103 highly myopic eyes (≥ −6.00 dioptres) and 227 normal eyes randomly selected from the baseline population, matched for age and sex.

**Results:**

The mean SFCT was 110.6 ± 85.2 μm in highly myopic eyes (range, 3–395 μm). Mean regional choroidal thickness was lowest on the nasal and inferior sides of the macula, and slightly higher on the temporal and superior sides than at the fovea. On multivariate analysis, SFCT was associated with age (*b* = −0.48; *P* < 0.001), axial length (*b* = −0.44; *P* < 0.001), gender (*b* = −0.31; *P* < 0.05) and staphyloma (*b* = −0.26; *P* = 0.05). In highly myopic eyes, SFCT decreased by 5.1 μm/year of age, by 9.2 µm/D of myopia, and by 22.6 µm/mm of axial length.

**Conclusions:**

The SFCT decreases with age and increased axial length in highly myopic eyes. The formation of a posterior staphyloma has been identified as a major contributor to choroidal thinning and is therefore a reliable indicator for risk management. The involvement of choroidal abnormalities may be a significant factor in the development of myopic degeneration.

Myopia is the leading cause of visual impairment on a global scale [1]. The incidence of high myopia varies according to race and region [2]. By 2050, it is expected that almost half of the world’s population will be myopic and around 9.8% will be highly myopic [3].

High myopia is defined as a refractive error of −5.00 dioptres (D) or worse [4]. As the dioptre increases and the eyeball progressively extends, various degenerative changes occur in the fundus, including lacquer cracks in the Bruch membrane, posterior staphyloma, choroidal neovascularization (CNV), and chorioretinal atrophy [4]. Based on previous research, pathologic myopia was defined as the presence of myopic lesions in the posterior segment of the eye (posterior staphyloma or myopic maculopathy equal to or more serious than diffuse choroidal atrophy), which usually occurs when the refractive error is >6.0 D or the axial length of the eye is >26 mm [4]. However, the latest research indicates that refractive error or axial length alone does not fully characterise pathological myopia. For instance, posterior staphyloma is a hallmark lesion of pathological myopia, although it can also manifest in non-high refractive error eyes [5, 6].

Histological and imaging studies have suggested that the choroid is thinner in cases of high myopia, accompanied by the loss of choroidal capillaries in certain areas [7, 8]. The retinal dysfunction and vision loss observed in high myopia may be partly explained by the choroidal circulation or choroidal blood flow, as the choroid receives ~95% of the ophthalmic artery blood, which provides oxygen and supplies nutrition to the outer retinal layers [9].

Enhanced depth imaging spectral-domain optical coherence tomography (EDI SD-OCT) has supported a way to detect choroidal thickness in vivo [10]. Its deep-enhanced mode is more advantageous for displaying the structures of the outer retina and choroid, but the disadvantage is that it is not conducive to detailed resolution of the inner retina. Several studies have reported the subfoveal choroidal thickness (SFCT) in normal subjects and patients with choroidal or retinal diseases, including high myopia [11–14]. However, these studies were hospital-based studies, which is likely to introduce selection bias in the study population. Therefore, we attempt to measure the SFCT in eyes with high myopia using EDI SD-OCT in a population-based real-world study, and to identify the risk factors for the development of choroidal thinning in high myopia.

## Methods

### Inclusion criteria

The Beijing Eye Study is a population-based prospective cohort study in Northern China with an age of 50+ years. The study has been described in detail [15–17].

Based on the data from the Beijing Eye Study 2011, we conducted a cross-sectional study of highly myopic patients (defined as having a spherical equivalent (SE) refractive error equal to or more negative than −6 D). To reduce the influence of other eye-related diseases on choroidal thickness, we excluded patients with amblyopia, glaucoma, severe cataract, uveitis, diabetic retinopathy, moderate-to-severe choroidal retinal atrophy, retinal vascular anomalies, laser treatments, hyper-pigmentation, severe choroidal retinal atrophy, macular scarring, refractive surgery, intraocular surgery, and patients after vitrectomy. Furthermore, patients with pathological structures, such as choroidal neovascularization foveoschisis, macular pucker and macular hole, which could affect choroidal thickness on SD-OCT, were also excluded.

Of the total 3468 subjects, high myopia was detected in 135 (1.95%) eyes (prevalence rate (mean ± SE): 1.95 ± 0.17%; 95% CI: 1.62%, 2.27%) of 93 (2.68%) subjects (prevalence rate: 2.68 ± 0.27%; 95% CI: 2.14%, 3.22%). Optical coherence tomography (OCT) images of sufficient quality for analysis were available for 103 eyes of 70 (70.0%) participants (50 (64.1%) women). In 15 (16.1%) subjects, OCT images could not be analysed either because no images were taken or because the available images could not be evaluated due to lens opacities or vitreous opacities. Compared to the highly myopic subjects with OCT examination, the group without examination was significantly older (71.3 ± 7.7 years versus 64.8 ± 8.5 years; *P* = 0.007; 95% CI: 1.87, 11.29), but did not differ significantly in gender (*P* = 0.85) or refractive error (*P* = 0.67).

As a control, 227 normal eyes of 227 individuals were randomly selected from the base, and matched for age and sex to the population. In selecting the control group, the above-mentioned diseases that may affect choroidal thickness were also excluded.

### Basic examination

The research protocol was approved by the Ethics Committee. All examinations were conducted in the communities, either in school buildings or in community houses. After obtaining informed consent, an interview was conducted with standardised questions about marital status, educational level, income, quality of life, psychological depression, physical activity, known major systemic diseases such as arterial hypertension and diabetes mellitus, and quality of vision. Fasting blood samples were taken for measurement of blood lipids, glucose, and glycosylated haemoglobin HbA1c. Blood pressure was measured. Height, weight, and waist and hip circumference were recorded. The ophthalmic examination included measurement of presenting visual acuity and uncorrected visual acuity. Best-corrected visual acuity was assessed by automated refractometry (Auto Refractometer AR-610, Nidek Co., Ltd., Tokyo, Japan). If uncorrected visual acuity was <1.0, subjective refraction was also performed. Intraocular pressure was measured by pneumotonometry by an experienced ophthalmologist. Corneal disorders and peripheral anterior chamber depth according to the van Herick method were assessed by slit-lamp examination by an ophthalmologist. The anterior segment was measured by slit-lamp adapted OCT (Heidelberg Engineering Co., Dossenheim, Germany). Low-coherence optical reflectometry (Lensstar 900® Optical Biometer, Haag-Streit, 3098 Koeniz, Switzerland) was used to measure anterior corneal curvature, central corneal thickness, anterior chamber depth, lens thickness, and axial length in the right eye (or left eye if right eye measurements were not possible). The pupil was dilated with tropicamide until the pupil diameter was at least 6 mm. Digital photographs of the cornea and lens were taken with a slit-lamp digital camera (type BG-4, Topcon Medical Systems, Inc., Tokyo, Japan), and retro illuminated photographs of the lens were taken with a Neitz CT-R camera (Neitz Instruments Co., Tokyo, Japan). Photographs of the macula and optic disc were taken with a fundus camera (type CR6-45NM, Canon Inc., USA), and the colour fundus photographs were used to observe myopic degeneration, including macular atrophy and haemorrhages, by two independent observers.

### Measurement of choroidal/retinal parameters

SFCT was measured using SD-OCT (Spectralis®, wavelength: 870 nm; Heidelberg Engineering Co., Heidelberg, Germany) with the EDI modality. The instrument was positioned close enough to the eye with an undilated pupil to produce an inverted image. Seven sections, each consisting of 100 averaged scans, were obtained in a 5°–30° rectangle centred on the fovea, with a single-line scan range of 3–10 mm. The horizontal section through the centre of the fovea was selected for further analysis. Mean regional thickness was calculated for five scan lines of 55 points, including superior, inferior, temporal, and nasal quadrants. These measurements were taken from the subfoveal choroid and at 500 μm intervals from the fovea to 2.5 mm nasal and 2.5 mm temporal from the centre of the fovea. SFCT was defined as the vertical distance from the hyperreflective line of Bruch’s membrane to the hyperreflective line of the inner surface of the sclera (Supplementary Fig. 1). Measurements were performed using the Heidelberg Eye Explorer software (version 5.3.3.0; Heidelberg Engineering Co., Heidelberg, Germany). Measurements were performed independently by two examiners and interobserver reproducibility was found to be high (*r* = 0.992, *P* = 0.000). Briefly, the retinal thickness was measured by the same software, which was defined as the vertical distance from the retinal pigment epithelial (RPE) cells (the outermost hyperreflective line at the retina–choroid interface) to the retinal surface and the posterior staphyloma in relation to the horizontal plane was also evaluated in the OCT images. All measurements were taken on the same machine and in the same mode, with no individual magnification or reduction of the images. A posterior staphyloma is characterised by an outpouching of a defined region in the posterior fundus, with a curvature radius smaller than that of the adjacent eye wall. The presence of a posterior staphyloma can be determined on OCT based on the curvature of the choroid [6, 18].

### Statistical analysis

Statistical analysis was performed using commercially available statistical software (SPSS for Windows, version 25.0, SPSS, Chicago, IL, USA). SFCT of both normal and highly myopic eyes was described by means (presented as mean standard deviation). The results of the normality analysis of the SFCT are detailed in Supplementary Fig. 4. Categorical variables were assessed individually using the Chi-squared test, and the Fisher exact test was used for samples with an expectation of <5. Continuous data were analysed using an independent samples *t*-test. Repeated measures analysis of variance was used to analyse differences in choroidal thickness by macular location. Simple linear regression was calculated for variations in macular choroidal thickness in relation to systemic and ocular risk factors. Multiple linear regression was used to evaluate the explanatory variables in relation to the dependent variable. Only the right eye of each participant was included in the linear regression analysis. Ninety-five percent confidence intervals (CI) are presented. All *P* values were two-sided and considered statistically significant if the values were <0.05.

## Results

There was a total of 103 eyes in 70 patients in the high myopia group and 227 eyes in 227 patients in the control group. The mean age of patients was 64.5 ± 8.3 years (range, 50–85 years) in the highly myopic group and 65.2 ± 8.8 years (range, 50–82 years) in the control group (*P* = 0.464). The mean refractive error was −9.54 ± 3.57 D (range, −6.0 to −20.0 D) in the highly myopic group and 0.35 ± 0.67 D (range, −1.5 to 2.75 D) in the control group (*P* < 0.001). The average SFCT measured 110.6 ± 85.2 μm (range, 3–395 μm) in the highly myopic group and 263.0 ± 92.9 μm (range, 53–578 μm) in the control group (*P* < 0.001). Table 1 provides a summary of all baseline data.

**Table 1:** Comparison of demographic and ocular characteristics between highly myopic and normal eyes.

Factors	Mean ± SD		*P* (two-tailed)
	Highly myopic eye (*N* = 135)	Normal eye (*N* = 227)	
Age	64.5 ± 8.3	65.2 ± 8.8	0.471
Gender (male/female)	37/66	89/138	0.569^a^
Height (cm)	162.2 ± 9.5	161.2 ± 7.9	0.421
Weight (kg)	64.2 ± 12.7	66.2 ± 11.2	0.206
Best-corrected visual acuity	0.67 ± 0.29	1.04 ± 0.08	<0.001
Refractive error (D)	−9.54 ± 3.57	0.35 ± 0.67	<0.001
Axial length (mm)	26.62 ± 1.98	23.07 ± 0.72	<0.001
IOP (mmHg)	15.2 ± 2.5	14.5 ± 2.8	0.051
Subfoveal retinal thickness (μm)	208.9 ± 54.4	216.8 ± 17.5	0.149
Subfoveal choroidal thickness (μm)	110.6 ± 85.2	263.0 ± 92.9	<0.001

^a^The gender assessed individually with the Chi-square test, other factors assessed with independent sample *t*-test.

**Fig. 1:**
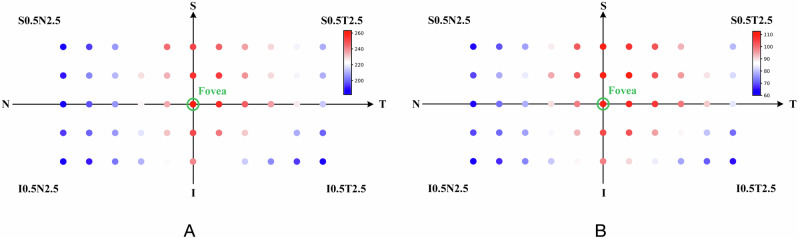
Average choroidal thickness and thickness distribution map of 55 locations. **A** The average thickness and distribution of the choroid in a normal eye. **B** The average thickness and distribution of the choroid in a highly myopic eye.

Figure 1 shows the mean choroidal thickness and thickness distribution for the 55 points. In healthy eyes, choroidal thickness was greatest at the fovea with a mean of 263.0 ± 92.9 μm. Away from the fovea, choroidal thickness decreased rapidly nasally and inferiorly, reaching a minimum of 182.2 ± 89.7 μm at 2.5 mm nasal and 0.5 mm inferior to the fovea (Fig. 1 and Supplementary Table 1). A notable difference was observed between subfoveal choroidal depth and depth at other extrafoveal locations (Supplementary Table 1). In highly myopic eyes, all 55 measurement points were significantly thinner than the corresponding points in normal eyes. Regional choroidal thickness was lowest at the nasal and inferior macula at 60.0 ± 50.7 μm. Thickness at the temporal and superior regions was slightly greater than at the fovea (Supplementary Table 2).

Table 2 shows the results of the univariate analysis, SFCT in highly myopic eyes was significantly associated with the systemic parameters of younger age (*P* < 0.001), male gender (*P* = 0.008), higher serum glucose concentrations (*P* = 0.002); with the ocular parameters of shorter axial length (*P* < 0. 001), positive refractive error (*P* < 0.001), higher best-corrected visual acuity (*P* = 0.024), higher ocular perfusion pressure (*P* = 0.027), thicker subfoveal retinal thickness (*P* = 0.021); and with the pathological factors of myopia, absence of macular atrophy (*P* < 0.001) and staphyloma (*P* < 0.001). It was not significantly associated (all *P* > 0.05) with the systemic parameters of height, weight, systolic and diastolic blood pressure, presence of diabetes mellitus, smoking and alcohol consumption, and aspirin use. In addition, SFCT in highly myopic eyes did not correlate with ocular parameters such as central corneal thickness, anterior chamber depth, lens thickness, corneal curvature and diameter, pupil diameter, or pathological myopic factors of macular haemorrhage.

**Table 2:** Univariate associations between SFCT with highly myopic eyes (as measured by enhanced depth imaging of spectral-domain optical coherence tomography) and ocular and general parameters in the Beijing Eye Study 2011.

Parameter	Unstandardised coefficients (B)	95% confidence interval	Standardised coefficients (beta)	*P* value
Systemic parameters				
Age (years)	−5.06	−6.81, −3.32	−0.50	<0.001
Gender	−45.63	−79.31, −11.95	−0.26	0.008
Body height (cm)	1.95	−0.23, 4.14	0.21	0.080
Body weight (kg)	1.47	−0.18, 3.11	0.21	0.080
Systolic blood pressure (mmHg)	−0.64	−1.58, 0.29	−0.16	0.180
Diastolic blood pressure (mmHg)	0.83	−0.84, 2.50	0.12	0.990
Glucose (mmol/l)	23.53	8.97, 38.08	0.45	0.002
Diabetes mellitus	8.76	−61.27, 78.78	0.03	0.803
Smoking	19.00	−10.69, 48.69	0.16	0.206
Alcohol consumption	13.66	−0.87, 28.20	0.23	0.065
Aspirin intake	−38.08	−81.20, 5.03	−0.22	0.082
Ocular parameters				
Axial Length (mm)	−22.56	−32.39, −12.74	−0.51	<0.001
Refractive error (D)	9.18	4.84, 13.52	0.39	<0.001
Best corr. visual acuity (logMAR)	81.65	11.13, 152.17	0.27	0.024
Ocular perfusion pressure (mmHg)	7.34	0.87, 13.81	0.22	0.027
Subfoveal retinal thickness (µm)	0.36	0.06, 0.66	0.23	0.021
Centre corneal thickness (µm)	0.26	−0.34, 0.91	0.10	0.236
Anterior chamber depth (mm)	18.02	−28.19, 64.23	0.10	0.438
Lens thickness (mm)	−57.51	−120.55, 5.53	−0.24	0.073
Corneal curvature (mm)	−30.79	−105.54, 43.97	−0.11	0.413
Corneal diameter (mm)	5.30	−19.56, 30.17	0.06	0.671
Pupil diameter (mm)	1.08	−22.79, 24.94	0.01	0.928
Pathological myopic factors				
Macular atrophy	−79.52	−118.24, −40.79	−0.45	<0.001
Staphyloma	−102.85	−137.00, −68.71	−0.59	<0.001
Macular haemorrhage	−41.04	−98.02, 15.94	−0.17	0.155

A stepwise multiple linear regression analysis was then performed to identify the explanatory variables that had the strongest correlation with SFCT in highly myopic eyes. Age was the factor most associated with choroidal thickness (*b* = −0.48; *P* < 0.001), followed by axial length (*b* = −0.44; *P* < 0.001), gender (*b* = −0.31; *P* < 0.05) and staphyloma (*b* = −0.26; *P* = 0.05). Other factors were not significantly correlated with choroidal thickness (*P* all >0.05). Table 3 shows the results of the multiple regression analysis.

**Table 3:** Multivariate analysis of associations between SFCT in highly myopic eyes (as measured by enhanced depth imaging of spectral-domain optical coherence tomography) and ocular and general parameters in the Beijing Eye Study 2011.

Parameter	Unstandardised coefficients (B)	95% confidence interval	Standardised coefficients (beta)	*P* value
Staphyloma	−45.35	−90.81, 0.11	−0.26	0.050
Age (years)	−5.12	−7.39, −2.85	−0.48	<0.001
Axial length (mm)	−19.75	−30.70, −8.81	−0.44	0.001
Gender	−60.32	−104.75, −15.89	−0.31	0.009

*R*^2^ = 0.63.

The univariate analysis indicates an association between high myopia SFCT and age (Supplementary Fig. 2). For each year increase in age, SFCT decreased by 5.1 μm (95% CI: −6.81, −3.32) (Table 2). If the whole study population was stratified into age groups of 10 years each, the decrease in SFCT per year of age did not vary markedly between the age group of 51–60 years (decrease in SFCT of 5.1 μm (95% CI: −16.7, 6.6, *P* = 0.380) per year of age), the age group of 61–70 years (3.3 μm (95% CI: −11.4, 4.8, *P* = 0.415)), and the age group of 70+ years (2.1 μm (95% CI: −8.8, 4.7, *P* = 0.535)). In the multivariate analysis, the decrease in SFCT per year of age was 5.1 μm (95% CI: −7.39, −2.85) (Table 3).

The regressions of the associations of SFCT of high myopia with axial length or of SFCT with refractive error showed a linear course (Supplementary Figs. 3 and 4). For the myopic refractive error range of more than −1 dioptres, it was highly significantly correlated (*P* < 0.001). For each 1 dioptre increase in myopic refractive error of one dioptre beyond a refractive error of −1 dioptre, SFCT decreased by 9.2 µm (95% CI: 4.84, 13.52). For the same range of refractive error, for every increase in axial length of 1 mm, SFCT decreased by 22.6 µm (95% CI: −32.39, −12.74) for each millimetre increase in axial length (Table 2).

Staphyloma was found to be the key factor influencing SFCT using stepwise multiple linear regression analysis. There were 32 eyes of 32 high myopic patients with staphyloma and 38 eyes of 38 patients without staphyloma with available OCT images. The mean SFCT was significantly lower in the high myopic group with staphyloma (54.94 ± 49.96 µm) compared to high myopic eyes without staphyloma (157.79 ± 85.18 µm) (*P* < 0.001). We attempted to identify an association between staphyloma and the development of SFCT thinning. All demographic and ocular data of the two groups are summarised in Table 4. Linear regression of the explanatory variables with SFCT reduced to a model including age in years in both the staphyloma group (*b* = −3.71, *P* = 0.001) and the no staphyloma group (*b* = −4.32, *P* = 0.003). Statistical results showed that axial length was significantly correlated with SFCT in the staphyloma group (*b* = −11.82, *P* = 0.011) but not in the no staphyloma group (*P* = 0.284); gender had no correlation with SFCT in highly myopic eyes with or without staphyloma (*P* = 0.286, *P* = 0.726).

**Table 4:** Comparison of demographic and ocular characteristics between highly myopia with and without staphyloma.

Factors	Mean ± SD		*P* (two-tailed)
	Staphyloma group	No staphyloma group	
Age	66.6 ± 8.2	63.8 ± 9.2	0.182
Gender (male/female)	7/25	19/19	0.015^a^
Height (cm)	158.4 ± 8.7	165.6 ± 9.5	0.001
Weight (kg)	60.3 ± 11.5	67.3 ± 13.1	0.017
Best-corrected visual acuity	0.56 ± 0.30	0.75 ± 0.26	0.006
Refractive error (D)	−11.61 ± 4.05	−7.48 ± 1.93	<0.001
Axial length (mm)	27.75 ± 1.77	25.70 ± 1.65	<0.001
IOP (mmHg)	14.6 ± 2.1	15.6 ± 3.2	0.136
Subfoveal retinal thickness (μm)	188.3 ± 53.5	214.9 ± 27.6	0.015
Subfoveal choroidal thickness (μm)	54.9 ± 50.0	157.8 ± 85.2	<0.001

^a^The gender assessed individually with the Chi-square test, other factors assessed with independent sample *t*-test.

## Discussion

In our real-world study on a relatively large population, we found that SFCT of EDI SD-OCT measurements indicated that the choroid of highly myopic eyes was significantly thinner at 110.6 ± 85.2 µm compared to normal eyes at 263.0 ± 92.9 µm (*P* < 0.001). The mean choroidal thickness measurement was thickest below the fovea in normal eyes and decreased rapidly nasally and inferiorly. Similarly, in highly myopic eyes, the mean regional choroidal thickness was thinnest at the nasal and inferior macula, whereas the thickness at the temporal and superior areas was slightly greater than that at the fovea (Supplementary Table 2). The results of multivariate analysis showed that SFCT in highly myopic eyes was associated with younger age, shorter axial length, male sex, and absence of staphyloma. Multiple linear regression analysis showed that SFCT in highly myopic eyes decreased by 5.1 µm/year of age, by 9.2 µm/D of myopia, and by 22.6 µm/mm of axial length.

The mean SFCT results for high myopia in our study differ somewhat from previous reports. Notably, the SFCT measurements in this study are marginally lower than those reported previously. Wang et al. performed a cross-sectional study and meta-analysis on choroidal thickness and high myopia [19]. Data from 301 eyes of 171 highly myopic patients were collected. The average age of highly myopic patients was 22.23 ± 6.50 years, with a mean SE of −7.56 ± 1.99 dioptres, average axial length (AL) of 26.56 ± 1.01 mm, and mean choroidal thickness in the macular region of 200.54 ± 69.39 μm. Deng et al. conducted a study on the distribution of choroid in Chinese myopic children [20], with a mean age of 11.93 ± 1.78 years. The average choroidal thickness in the macular region was 229 ± 65 μm.

This may be due to differences in the average age of the study population, the refractive error, and, perhaps, ethnic disparities in globe anatomy. The average age of our study population is 64.5 ± 8.3 years, with a refractive error of −9.54 ± 3.57 D and an axial length of 26.62 ± 1.98 mm. The study carried out by Wang et al. on 24 highly myopic individuals, with an average age of 35.5 years and SE of more than 8.00 D, demonstrated a mean SFCT of 123.7 μm, which can be compared to our findings if age and refractive degree variations are considered [21]. Accordingly, Ikono et al. measured the SFCT of highly myopic Japanese individuals with an average age of 51.7 years and reported a mean value of 100.5 μm [22]. This value is three times lower than our finding after adjusting for age. Similarly, in Fujiwara’s study on 55 highly myopic eyes with a mean age of 59.7 years, SFCT was 93.2 μm, which is thinner than our results [23].

The thickness of the choroid in normal eyes was found to be the greatest under the fovea in the regional choroidal scan of the posterior pole. A possible reason for this is that the inner retina gradually thins and disappears near the fovea, where the interconnected capillary network forms a single layer of vascular arches, leaving a foveal avascular zone with a diameter of ~450–500 μm [24]. The nutritional supply to this area mainly depends on the choriocapillaris, leading to a thicker choroid in the macular region. Angiographic studies have also indicated that the choroidal arteries and choriocapillaris in the macula fill more quickly and densely than in other areas of the retina [25]. In normal subjects, choroidal thickness is thickest below the macula and varies slightly in different directions: horizontally, the choroid is thinner nasally than temporally, and vertically, the choroid is thinner inferiorly than superiorly [12, 26].

The choroidal thickness distribution is similarly asymmetric in highly myopic eyes. The SFCT was similarly thinner than the nasal and inferior macula in highly myopic eyes, but thicker than the temporal and superior regions. The thinning of the foveal and nasal choroid may be related to the choroidal watershed often seen between the macula and the optic disc [27]. Thinning in the nasal area, particularly the peripapillary region, may increase the susceptibility to pathological choroidal thinning. Histologically, a reduction in choroidal thickness indicates a reduction in blood vessels within this layer. Clinically, choroidal thinning is thought to reflect a deficit in blood supply to this region. This vulnerability could potentially lead to peripapillary atrophy in pathological myopia. In addition, it suggests that chorioretinal atrophy in high myopia predominantly affects the fovea, nasal, and inferior regions rather than the superior and temporal regions.

The results of the stepwise multiple linear regression analysis indicate that age is the most significant factor linked to choroidal thickness. As the age advances, the choroidal vasculature is lost, leading to a decrease in choroidal perfusion. Remarkably, choroidal vascular inflammation due to aging was discovered in both the eyes of animals and humans [28, 29]. VAP-1 may induce the inflammatory response at a molecular level. The slowed metabolism of the outer retina due to choroidal thinning enhances the vascular inflammatory reaction [30].

This study demonstrates significant differences in the rate of choroidal thinning between normal and highly myopic eyes. The rate of choroidal thinning in highly myopic eyes (5.1 μm/year) is greater than the rate found in normal eyes (3.1 μm/year). This phenomenon is age-related [31]. In highly myopic eyes, pathological choroidal atrophy may accelerate the process, reducing the supply of oxygen and nutrients to the outer retina below the minimum required for normal visual function.

Parallel to age, axial length (or myopic refractive error) was a significant factor affecting SFCT. Prolonged axial elongation can induce scleral weakening in axial myopia. Studies in human eyes have found a correlation between long axial length and scleral thinning [32]. Recent studies have confirmed that scleral endoplasmic reticulum stress and the PERK/ATF6 pathway control axial elongation in a mouse model of myopia [33]. The dopaminergic system has been implicated in ocular growth regulation, which is a mediator of light-adaptive changes in retinal circuitry and in RPE physiology, and has preventive effects on the development of high myopia with prolonged axial length in rabbits and guinea pigs [34].

With a decrease in SFCT of 9.2 µm for every one dioptre increase in highly myopic refractive error of one dioptre, a myopic refractive error of −21 dioptres results in the choroid almost disappearing. However, as previous studies have shown an association between increased refractive error and increased axial length [35], we only include axial length in the multivariate analysis.

The analysis regarding the presence or absence of staphyloma indicates that the group without staphyloma demonstrated better BCVA and less myopia, had a shorter axial length and thicker subfoveal retina as well as a taller and even taller and fatter than those in the group with staphyloma. For the SFCT, the group without staphyloma (157.79 ± 85.18 μm) was almost twice the size of the group with staphyloma (54.94 ± 49.96 μm) (*P* < 0.001). Previous studies have shown that posterior staphyloma is a defining characteristic of high myopia, likely due to the larger posterior staphylomas generating greater inward vector forces, leading to detachment or splitting of the neural retina [34]. This is facilitated by vitreous cortical shrinkage, epiretinal membrane, or a rigid inner limiting membrane [36]. The height of posterior staphyloma correlates with various pathologies seen in high myopia-specific diseases, such as the degree of lacquer cracks, RPE defect, and choroidal atrophy [22]. Therefore, this novel parameter of posterior staphyloma height is a promising indicator for the management of choroidal thinning and posterior staphyloma formation. However, its correlation with choroidal thinning requires further investigation. In addition to choroidal thinning, scleral thinning and disarray of scleral collagen fibres is a key factor in the development of posterior staphyloma [37]. Scleral thinning involves not only passive stretching, but also tissue loss and redistribution [38], with a decrease in collagen production, an increase in collagen degradation, and changes in glycosaminoglycan content [37]. Changes in collagen fibre orientation and the accumulation of small-diameter fibres can be observed by electron microscopy [39].

There are some limitations to this study. First, there are measurement limitations. Currently, the average choroidal thickness measured by SD-OCT is relatively coarse, and detailed measuring method can be seen in refs. 40, 41. Recent studies have shown that it is possible to correct for the ocular magnification effect based on each participant’s biological characteristics and refractive measurements, resulting in more precise measurements of retinal and choroidal thickness [42, 43]. In the future we will refer to the latest methods to correct for the magnification of OCT images. Second, there are limitations in the statistical methods used. In the future, as the sample size increases, the use of multi-level modelling for statistical analysis will provide more accurate results.

In summary, the SFCT was significantly thinner in highly myopic eyes than in normal eyes. In high myopia, the SFCT decreased by 5.1 μm/year of age, by 9.2 µm/D of myopia, and by 22.6 µm/mm of axial length. Age, followed by axial length, gender, and posterior staphyloma, was most strongly associated with SFCT. These findings may indicate that abnormal changes in choroidal thickness play an important role in the pathogenesis of myopic degeneration, as SFCT is strongly correlated with staphyloma, age and axial length of the eye.

## Summary

### What was known before:

- Histological and imaging studies have suggested that the choroid is thinner in cases of high myopia, accompanied by the loss of choroidal capillaries in certain areas.

### What this study adds:

- This study measures the subfoveal choroidal thickness in highly myopic eyes at different locations using enhanced depth imaging spectral-domain optical coherence tomography.

## Supplementary information

Supplementary Figure 1-5

Supplementary Table 1-2

## Data Availability

The datasets generated and analysed during the current study are available from the corresponding author on reasonable request.
