# Supplementary material for: Distribution and associated factors of choroidal thickness in highly myopic eyes—a real-world study based on a Chinese population
Source: Eye (Lond). 2024 Oct 24;39(1):102–8. doi: 10.1038/s41433-024-03383-9 (PMC11733017; doi:10.1038/s41433-024-03383-9)
Supplement: Supplementary file 1 — Supplementary Figure 1-5 [file 41433_2024_3383_MOESM1_ESM.docx]

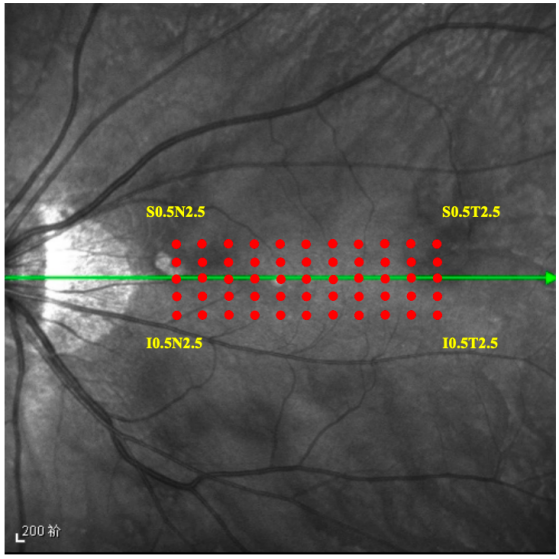


A


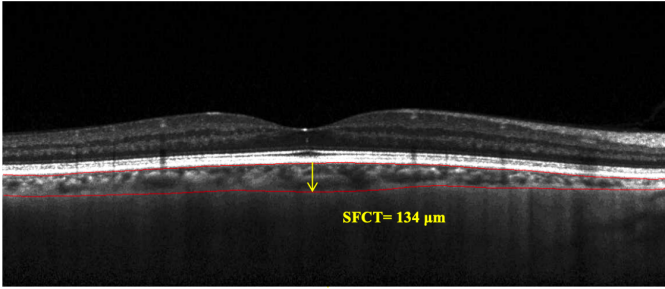


B


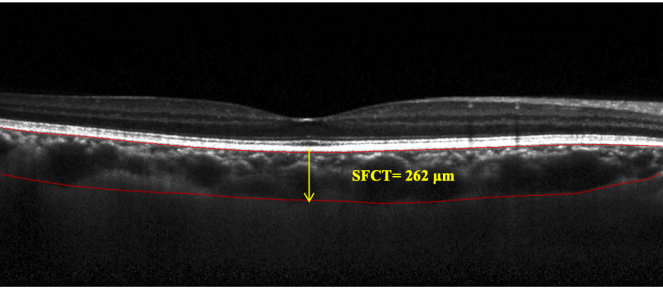


C

Sup Fig. 1 Schematic diagram depicting the enhanced depth-imaging optical coherence tomography (OCT) method for measuring choroidal thickness. A shows 55 points covering the superior, inferior, temporal and nasal quadrants. These measurements were taken at 500 μm intervals starting from fovea to 2.5 mm nasally and 2.5 mm temporally. B demonstrates measurement of SFCT in normal people eye. C demonstrates measurement of SFCT in highly myopic eye
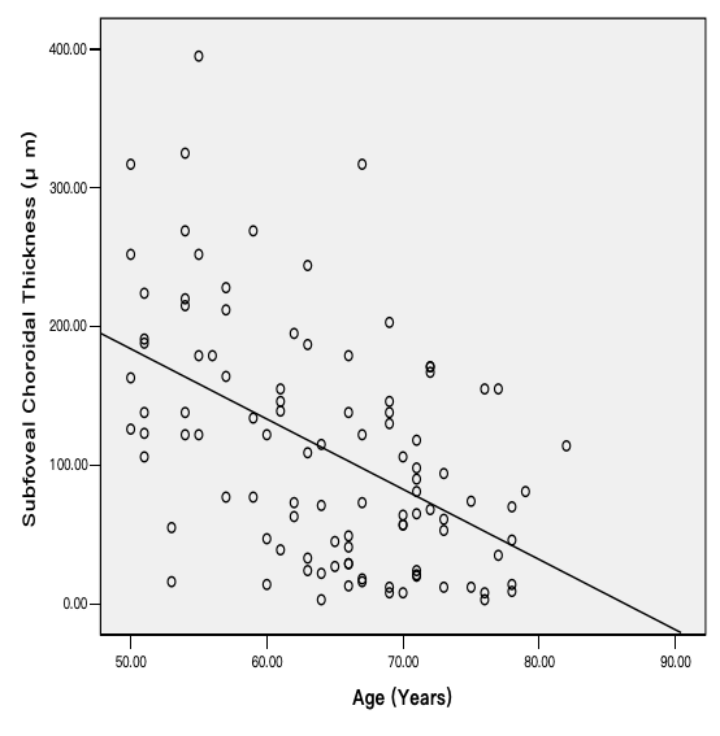


Sup Fig. 2 Scatterplot showing the distribution of subfoveal choroidal thickness (as measured by enhanced depth imaging of spectral domain optical coherence tomography) versus age in highly myopic eyes (P<0.001; Y = -5.1X +437.2; R^2^= 0.25)


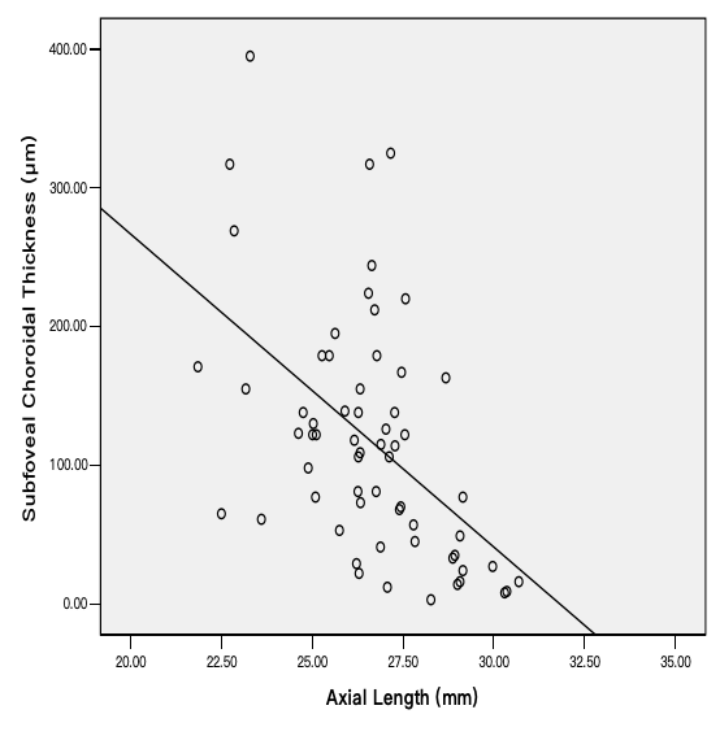


Sup Fig. 3 Scatterplot showing the distribution of subfoveal choroidal thickness (as measured by enhanced depth imaging of spectral domain optical coherence tomography) versus axial length in highly myopic eyes (P<0.001; Y = -22.6X +717.9; R^2^= 0.26)


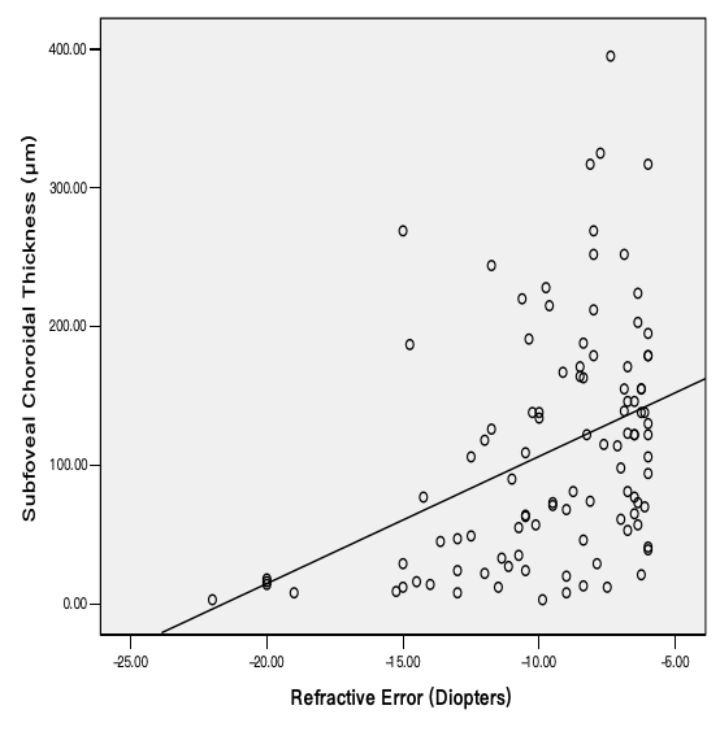


Sup Fig. 4 Scatterplot showing the distribution of subfoveal choroidal thickness (as measured by enhanced depth imaging of spectral domain optical coherence tomography) versus refractive error in highly myopic eyes (P<0.001; Y= 9.2 X+ 198.2; R^2^=0.15)


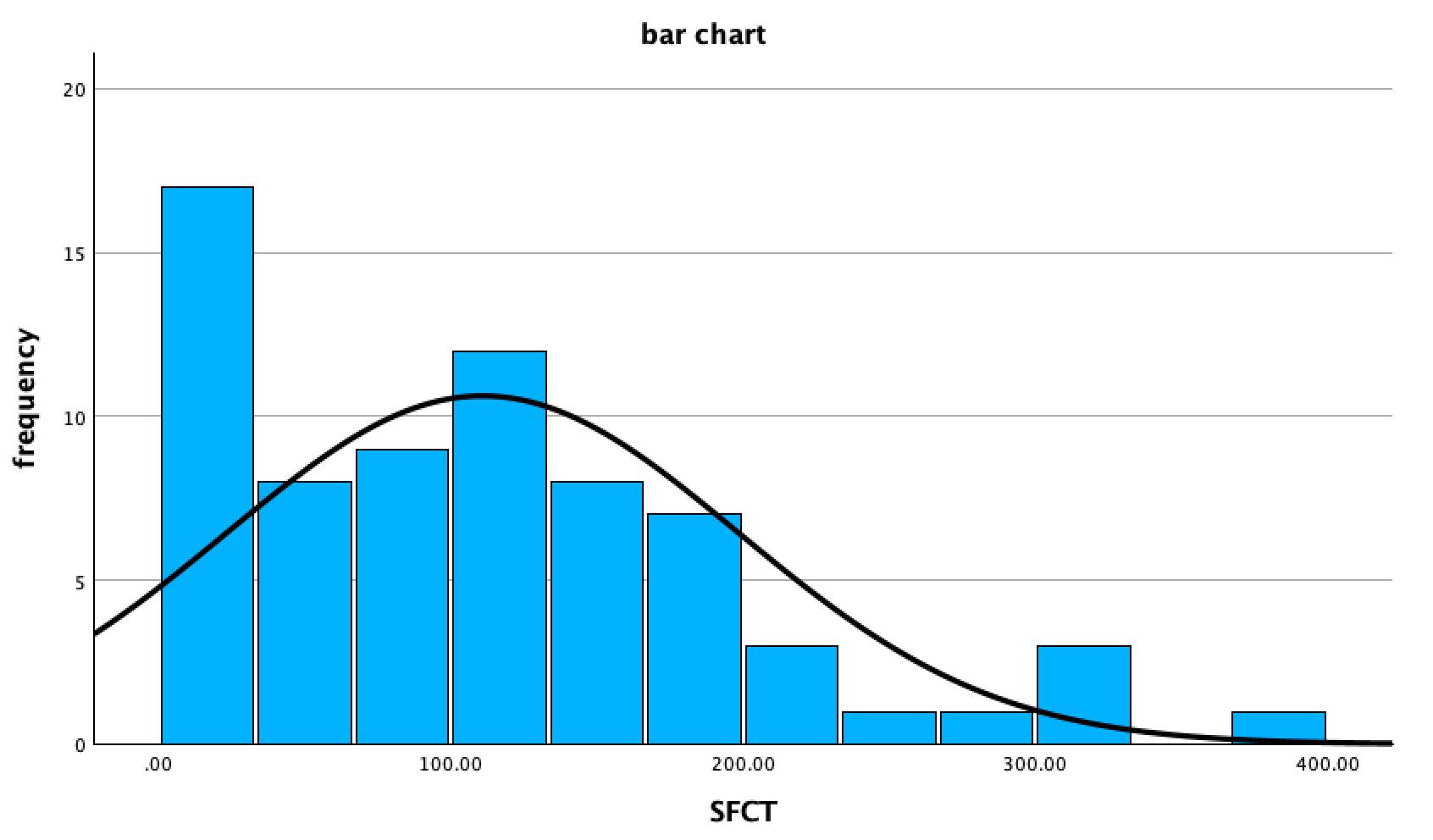


A


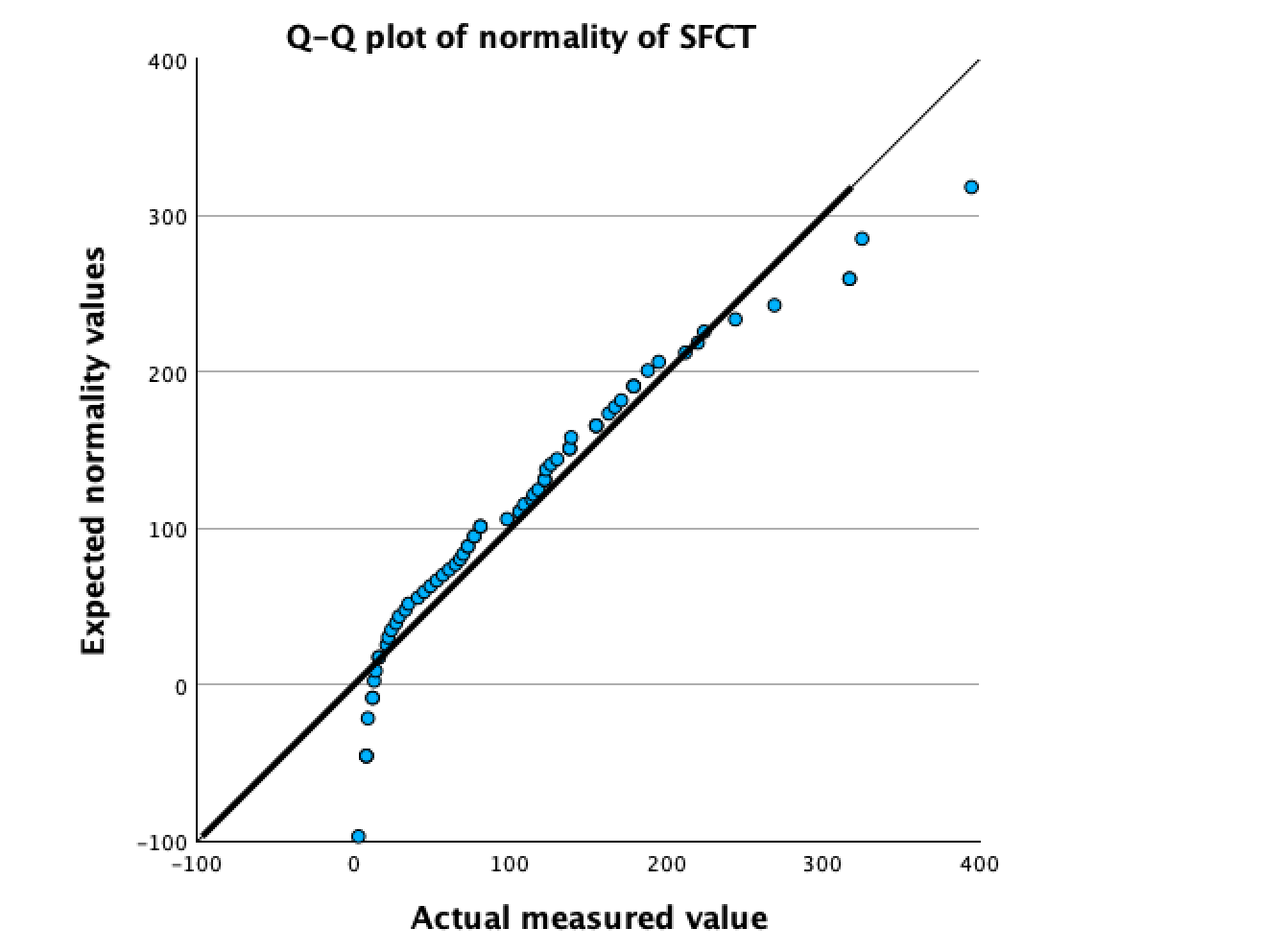


B

Sup Fig. 5 Normality test for SFCT measurements. (A) Bar chart of SFCT measurements. (B) Q-Q plot of normality of SFCT measurements.
