# Supplementary material for: Distribution and associated factors of choroidal thickness in highly myopic eyes—a real-world study based on a Chinese population
Source: Eye (Lond). 2024 Oct 24;39(1):102–8. doi: 10.1038/s41433-024-03383-9 (PMC11733017; doi:10.1038/s41433-024-03383-9)
Supplement: Supplementary file 2 — Supplementary Table 1-2 [file 41433_2024_3383_MOESM2_ESM.docx]

Supplement TABLE 1. Mean Choroidal Thickness Measurements at Various Locations in normal eyes.

| Location(mm) | Mean Choroidal Thickness (μm) | Standard Error | Difference in Mean Choroidal Thickness  (compared to Fovea) | Standard Error | P value |
| --- | --- | --- | --- | --- | --- |
| Fovea | 263.0 | 6.2 |  |  |  |
| SFN0.5 ^a^ | 234.9 | 6.1 | 28.1 | 0.3 | <0.001 |
| SFN1.0 | 224.6 | 6.0 | 38.4 | 0.4 | <0.001 |
| SFN1.5 | 206.4 | 6.0 | 56.6 | 0.8 | <0.001 |
| SFN2.0 | 196.8 | 6.0 | 66.2 | 0.7 | <0.001 |
| SFN2.5 | 184.8 | 6.0 | 78.2 | 0.9 | <0.001 |
| SFT0.5 | 256.5 | 6.1 | 6.5 | 0.3 | <0.001 |
| SFT1.0 | 246.3 | 6.2 | 16.7 | 0.7 | <0.001 |
| SFT1.5 | 236.8 | 6.1 | 26.2 | 0.3 | <0.001 |
| SFT2.0 | 226.3 | 6.0 | 36.7 | 0.4 | <0.001 |
| SFT2.5 | 214.0 | 6.0 | 49.0 | 0.6 | <0.001 |
| S0.25SF | 254.9 | 6.1 | 8.1 | 0.4 | <0.001 |
| S0.25N0.5 | 234.1 | 6.0 | 28.9 | 0.5 | <0.001 |
| S0.25N1.0 | 227.8 | 6.0 | 35.2 | 0.5 | <0.001 |
| S0.25N1.5 | 211.5 | 5.9 | 51.5 | 0.7 | <0.001 |
| S0.25N2.0 | 204.0 | 5.9 | 59.0 | 0.7 | <0.001 |
| S0.25N2.5 | 190.0 | 5.9 | 72.9 | 0.7 | <0.001 |
| S0.25T0.5 | 252.6 | 6.0 | 10.4 | 0.4 | <0.001 |
| S0.25T1.0 | 238.2 | 6.0 | 24.8 | 0.5 | <0.001 |
| S0.25T1.5 | 228.9 | 6.0 | 34.1 | 0.5 | <0.001 |
| S0.25T2.0 | 220.4 | 6.0 | 42.6 | 0.7 | <0.001 |
| S0.25T2.5 | 210.6 | 6.0 | 52.4 | 0.7 | <0.001 |
| S0.5SF | 251.0 | 6.1 | 11.9 | 0.8 | <0.001 |
| S0.5N0.5 | 243.9 | 6.1 | 19.1 | 0.3 | <0.001 |
| S0.5N1.0 | 222.4 | 6.0 | 40.6 | 0.6 | <0.001 |
| S0.5N1.5 | 206.9 | 6.1 | 56.1 | 0.8 | <0.001 |
| S0.5N2.0 | 194.4 | 6.0 | 68.6 | 0.6 | <0.001 |
| S0.5N2.5 | 186.1 | 6.0 | 76.9 | 0.6 | <0.001 |
| S0.5T0.5 | 245.5 | 6.1 | 17.5 | 0.4 | <0.001 |
| S0.5T1.0 | 236.4 | 6.1 | 26.6 | 0.4 | <0.001 |
| S0.5T1.5 | 229.2 | 6.1 | 33.8 | 0.4 | <0.001 |
| S0.5T2.0 | 220.2 | 6.1 | 42.8 | 0.4 | <0.001 |
| S0.5T2.5 | 210.1 | 6.0 | 52.9 | 0.5 | <0.001 |
| I0.25SF | 249.8 | 6.1 | 13.2 | 0.3 | <0.001 |
| I0.25N0.5 | 231.5 | 6.0 | 31.5 | 0.3 | <0.001 |
| I0.25N1.0 | 217.9 | 6.0 | 45.1 | 0.4 | <0.001 |
| T0.25N1.5 | 207.0 | 6.0 | 56.0 | 0.5 | <0.001 |
| I0.25N2.0 | 199.0 | 6.0 | 64.0 | 0.6 | <0.001 |
| I0.25N2.5 | 193.9 | 6.0 | 69.1 | 0.5 | <0.001 |
| I0.25T0.5 | 242.3 | 6.1 | 20.7 | 0.2 | <0.001 |
| I0.25T1.0 | 230.6 | 6.1 | 32.3 | 0.3 | <0.001 |
| I0.25T1.5 | 222.0 | 6.1 | 41.0 | 0.6 | <0.001 |
| I0.25T2.0 | 210.1 | 6.0 | 52.9 | 0.4 | <0.001 |
| I0.25T2.5 | 199.1 | 6.0 | 63.9 | 0.7 | <0.001 |
| I0.5SF | 240.3 | 6.1 | 22.6 | 0.3 | <0.001 |
| I0.5N0.5 | 221.1 | 6.0 | 41.9 | 0.5 | <0.001 |
| I0.5N1.0 | 211.2 | 6.0 | 51.8 | 0.5 | <0.001 |
| I0.5N1.5 | 201.8 | 6.0 | 61.2 | 0.6 | <0.001 |
| I0.5N2.0 | 190.5 | 6.0 | 72.5 | 0.6 | <0.001 |
| I0.5N2.5 | 182.2 | 6.0 | 80.7 | 0.6 | <0.001 |
| I0.5T0.5 | 222.7 | 6.0 | 40.3 | 0.5 | <0.001 |
| I0.5T1.0 | 214.7 | 5.9 | 48.2 | 0.6 | <0.001 |
| I0.5T1.5 | 204.7 | 6.0 | 58.3 | 0.5 | <0.001 |
| I0.5T2.0 | 193.9 | 5.9 | 69.1 | 0.6 | <0.001 |
| I0.5T2.5 | 186.9 | 6.0 | 76.1 | 0.8 | <0.001 |

^a^ Denotes the position 0.5 mm nasal to the fovea. The same naming convention is used for the subsequent entries.

Supplement TABLE 2. Mean Choroidal Thickness Measurements at Various Locations in highly myopic eyes.

| Location(mm) | Mean Choroidal Thickness (μm) | Standard Error | Difference in Mean Choroidal Thickness  (compared to Fovea) | Standard Error | P value |
| --- | --- | --- | --- | --- | --- |
| Fovea | 110.6 | 8.4 |  |  |  |
| SFN0.5 ^a^ | 98.6 | 7.4 | 12.0 | 1.9 | <0.001 |
| SFN1.0 | 89.8 | 7.1 | 20.8 | 1.9 | <0.001 |
| SFN1.5 | 78.3 | 6.6 | 32.3 | 2.5 | <0.001 |
| SFN2.0 | 72.0 | 6.4 | 38.6 | 2.8 | <0.001 |
| SFN2.5 | 63.6 | 6.2 | 47.0 | 3.2 | <0.001 |
| SFT0.5 | 109.1 | 7.9 | 1.5 | 1.6 | 0.363 |
| SFT1.0 | 106.4 | 7.6 | 4.2 | 1.4 | 0.004 |
| SFT1.5 | 99.4 | 7.3 | 11.2 | 1.7 | <0.001 |
| SFT2.0 | 91.5 | 7.1 | 19.1 | 2.0 | <0.001 |
| SFT2.5 | 83.6 | 6.8 | 27.0 | 2.3 | <0.001 |
| S0.25SF | 111.1 | 8.0 | -0.5 | 1.3 | 0.682 |
| S0.25N0.5 | 102.5 | 7.2 | 10.0 | 2.1 | <0.001 |
| S0.25N1.0 | 94.4 | 7.0 | 16.2 | 2.3 | <0.001 |
| S0.25N1.5 | 84.2 | 6.7 | 26.4 | 2.7 | <0.001 |
| S0.25N2.0 | 78.2 | 6.5 | 32.4 | 2.8 | <0.001 |
| S0.25N2.5 | 68.8 | 6.2 | 41.8 | 3.3 | <0.001 |
| S0.25T0.5 | 112.6 | 7.7 | -2.0 | 1.5 | 0.185 |
| S0.25T1.0 | 102.8 | 7.3 | 7.8 | 1.9 | <0.001 |
| S0.25T1.5 | 95.8 | 7.3 | 14.8 | 2.0 | <0.001 |
| S0.25T2.0 | 89.3 | 7.0 | 21.3 | 2.2 | <0.001 |
| S0.25T2.5 | 82.0 | 6.7 | 28.6 | 2.5 | <0.001 |
| S0.5SF | 110.9 | 7.6 | -0.3 | 1.5 | 0.825 |
| S0.5N0.5 | 102.5 | 7.5 | 8.1 | 1.5 | <0.001 |
| S0.5N1.0 | 88.5 | 7.1 | 22.1 | 2.0 | <0.001 |
| S0.5N1.5 | 77.0 | 6.7 | 33.7 | 2.5 | <0.001 |
| S0.5N2.0 | 69.5 | 6.4 | 41.1 | 2.8 | <0.001 |
| S0.5N2.5 | 63.8 | 6.2 | 46.8 | 3.1 | <0.001 |
| S0.5T0.5 | 106.9 | 7.4 | 3.7 | 2.1 | 0.084 |
| S0.5T1.0 | 103.1 | 7.6 | 7.5 | 1.5 | <0.001 |
| S0.5T1.5 | 93.6 | 7.2 | 17.0 | 1.8 | <0.001 |
| S0.5T2.0 | 86.5 | 7.1 | 24.1 | 2.0 | <0.001 |
| S0.5T2.5 | 79.3 | 6.8 | 31.3 | 2.4 | <0.001 |
| I0.25SF | 104.2 | 7.9 | 6.4 | 1.3 | <0.001 |
| I0.25N0.5 | 93.3 | 7.3 | 17.3 | 1.7 | <0.001 |
| I0.25N1.0 | 85.2 | 7.1 | 25.4 | 2.2 | <0.001 |
| I0.25N1.5 | 76.8 | 6.7 | 33.8 | 2.4 | <0.001 |
| I0.25N2.0 | 71.6 | 6.5 | 39.0 | 2.7 | <0.001 |
| I0.25N2.5 | 68.8 | 6.3 | 46.0 | 2.9 | <0.001 |
| I0.25T0.5 | 103.2 | 7.5 | 7.4 | 1.8 | <0.001 |
| I0.25T1.0 | 95.3 | 7.2 | 15.4 | 2.0 | <0.001 |
| I0.25T1.5 | 87.7 | 7.0 | 22.9 | 2.2 | <0.001 |
| I0.25T2.0 | 80.1 | 6.8 | 30.6 | 2.4 | <0.001 |
| I0.25T2.5 | 71.9 | 6.5 | 38.7 | 2.7 | <0.001 |
| I0.5SF | 98.5 | 7.6 | 12.1 | 1.4 | <0.001 |
| I0.5N0.5 | 87.7 | 7.1 | 22.9 | 2.0 | <0.001 |
| I0.5N1.0 | 80.7 | 6.8 | 29.9 | 2.3 | <0.001 |
| I0.5N1.5 | 74.0 | 6.7 | 36.7 | 2.5 | <0.001 |
| I0.5N2.0 | 66.7 | 6.4 | 43.9 | 2.9 | <0.001 |
| I0.5N2.5 | 60.0 | 6.2 | 50.7 | 3.2 | <0.001 |
| I0.5T0.5 | 90.6 | 7.9 | 20.1 | 2.1 | <0.001 |
| I0.5T1.0 | 84.4 | 6.8 | 26.2 | 2.3 | <0.001 |
| I0.5T1.5 | 77.3 | 6.7 | 33.3 | 2.7 | <0.001 |
| I0.5T2.0 | 70.3 | 6.4 | 40.3 | 3.0 | <0.001 |
| I0.5T2.5 | 64.6 | 6.2 | 46.0 | 3.2 | <0.001 |

^a^ Denotes the position 0.5 mm nasal to the fovea. The same naming convention is used for the subsequent entries.
